# Pathogenesis and Antifungal Drug Resistance of the Human Fungal Pathogen *Candida glabrata*

**DOI:** 10.3390/ph4010169

**Published:** 2011-01-11

**Authors:** Michael Tscherner, Tobias Schwarzmüller, Karl Kuchler

**Affiliations:** Medical University Vienna, Christian Doppler Laboratory for Infection Biology, Max F. Perutz Laboratories, Campus Vienna Biocenter, Dr. Bohr-Gasse 9/2, A-1030 Vienna, Austria; E-Mails: michael.tscherner@meduniwien.ac.at (M.T.); tobias.schwarzmuller@meduniwien.ac.at (T.S.)

**Keywords:** *Candida glabrata*, fungal pathogenesis, virulence, drug resistance

## Abstract

*Candida glabrata* is a major opportunistic human fungal pathogen causing superficial as well as systemic infections in immunocompromised individuals and several other patient cohorts. *C. glabrata* represents the second most prevalent cause of candidemia and a better understanding of its virulence and drug resistance mechanisms is thus of high medical relevance. In contrast to the diploid dimorphic pathogen *C. albicans,* whose ability to undergo filamentation is considered a major virulence trait, *C. glabrata* has a haploid genome and lacks the ability to switch to filamentous growth. A major impediment for the clinical therapy of *C. glabrata* infections is its high intrinsic resistance to several antifungal drugs, especially azoles. Further, the development of antifungal resistance, particularly during prolonged and prophylactic therapies is diminishing efficacies of therapeutic interventions. In addition, *C. glabrata* harbors a large repertoire of adhesins involved in the adherence to host epithelia. Interestingly, genome plasticity, phenotypic switching or the remarkable ability to persist and survive inside host immune cells further contribute to the pathogenicity of *C. glabrata*. In this comprehensive review, we want to emphasize and discuss the mechanisms underlying virulence and drug resistance of *C. glabrata*, and discuss its ability to escape from the host immune surveillance or persist inside host cells.

## 1. Introduction

*Candida* species are currently the fourth-leading cause of hospital-acquired bloodstream infections, reaching a mortality rate of up to ∼35–40% for systemic or disseminated infections [1,2]. Systemic mycoses can occur in patients with severely impaired immune systems (AIDS), people with organ or bone marrow transplants, cancer patients undergoing chemotherapy or in intensive care unit (ICU) patients, as well as both neonates and the elderly. The high mortality observed with systemic candidemia can be explained at least in part by a lack of fast and accurate diagnostic tools and in some cases by inefficient antifungal therapies. Therefore, there is a need for basic as well as clinical research to understand the molecular mechanisms of pathogenicity, to define the pathways and genetic networks driving the transition from commensalism (*i.e.* colonization) to host dissemination, and to develop novel antifungal drugs and diagnostic tools in order to improve treatment of fungal infections, especially those caused by *C. glabrata*.

Among all *Candida* species *C albicans* is still the most frequently isolated species, followed by *C. glabrata* accounting for ∼15–20% in Europe and ∼20% in North America of all clinical *Candida* spp isolates [1,3,4]. When compared to *C. albicans,* relatively little is known about the molecular mechanisms enabling *C. glabrata* to become a successful human pathogen. The genome organization indicates a synteny relationship to the well-known model non-pathogenic baker's yeast *Saccharomyces cerevisiae*. However, although haploid, *C. glabrata* lacks a sexual cycle and mating has never been observed. Moreover, prominent important virulence factors operating in *C. albicans* such as the formation of true hyphae, are absent in *C. glabrata* yet it managed to become a successful human pathogen. In this review, we want to summarize recent progress in the identification and characterization of different virulence factors and drug resistance mechanisms of *C. glabrata* (Table 1). For space constraints, we will limit this review to *C. glabrata*, but would like to refer to numerous excellent recent and comprehensive reviews addressing the pathobiology of *C. albicans* [5-10].

## 2. Adherence

Adherence to host cells and tissues is considered as a key virulence factor of many human fungal pathogens. Members of the *ALS* gene family encoding adhesins play a crucial role for interactions of *C. albicans* with host tissues [31,32]. In *C. glabrata*, the genome harbors a large group of putative GPI-anchored cell wall proteins [33], many of which are potential covalently-bound adhesins. The epithelial adhesin (*EPA*) gene family represents the largest group in *C. glabrata*, comprising at least 23 related genes, most of them located in subtelomeric regions [11,34]. The absolute number of *EPA* genes varies in different strain backgrounds and clinical isolates. For example, the BG2 strain contains 23, whereas the standard laboratory strain ATCC2001 (CBS138) strain carries only 17 *EPA* genes, lacking, for example, *EPA4* and *EPA5* [34,35]. The major epithelial adhesins, Epa1, Epa6 and Epa7, display different binding specificities concerning decoration of host cell ligands containing a terminal galactose residue [36]. Morover, the *C. glabrata* genome harbors a variety of additional putative adhesin families (Awp, Pwp), covalently surface-bound enzymatically active (Gas) or protein families of unknown function (Cwp, Pir). The presence of adhesin-like proteins (Awp1-4) in the cell surface strongly depends on the strain background and the growth phase [33,37].

*In vitro, C. glabrata* adherence to epithelial tissue is largely mediated by the major lectin Epa1, whereas other *EPA* genes are expressed at rather low level [11,12]. The adhesins *EPA6* and *EPA7* have been implicated in *C. glabrata* biofilm formation [13]. Epa6 seems to be a major player in biofilm formation, since it is highly induced during this phenomenon, and its absence reduces biofilms *in vitro*. Biofilms often typically display a higher resistance to several antifungal drugs. This is of special relevance, since *C. glabrata* naturally displays an inherent high azole resistance. Furthermore, *EPA6* expression is also induced by exposure to sorbic acid and parabens, which are used as preservatives in food and health products. The transcription factors Flo8 and Mss11 control weak organic acid stress induction of *EPA6*, leading to an increased adherence to vaginal epithelium due to the low pH in this environment [14].

The subtelomeric localization of most *EPA* genes places their expression under the control of the Sir-dependent chromatin silencing machinery [12]. In *C. glabrata*, this machinery depends on orthologoues of the *S. cerevisiae* silencing machinery, including Rap1, Sir2, Sir3, Sir4 and Rif1 [11,38]. For instance, expression of *EPA1*, *EPA6* and *EPA7* is induced in cells lacking the silencing genes *SIR3* and *RIF1*. In a murine model of disseminated candidiasis, *C. glabrata* silencing mutants are hyper-adherent to epithelial cells and more efficient in colonizing the kidney [12].

The transcriptional regulation of *EPA* gene expression is also controlled by host environmental signals such as limited nicotinic acid levels as present in the human urinary tract [15]. Interestingly, *C. glabrata* is an auxotroph for nicotinic acid (NA) and thus often causes urinary tract infections, since the low NA levels are sufficient to support *C. glabrata* growth. At the same time, the lack of NA, a precursor of NAD^+^ which is also a cofactor for the histone modifier Sir2, decreases Sir2 activity, resulting in the derepression of *EPA6*. In consequence, this leads to an increased adherence of *C. glabrata* to host tissues. Consistently, a triple *epa1*Δ *epa6*Δ *epa7*Δ mutant fails to colonize the bladder [15]. Notably, the NA auxotrophy of *C. glabrata* may actually reflect its close adaptation to the human host or even indicate adaptive co-evolution with the host, and enables *C. glabrata* to efficiently colonize a specific host niche.

Moreover, *C. glabrata* lacks most of the Biosynthesis of Nicotinic Acid (*BNA*) genes, and therefore must aquire any and all NAD^+^ precursors from its host environment. The NA uptake requires the membrane transporters Tna1, Tnr1 and Tnr2 [39]. In addition to NA, *C. glabrata* can utilize several different NAD^+^ precursors, including nicotinamide and nicotinamide riboside. During infections, nicotinamide riboside appears as the prime source of NAD^+^ [40]. Expression of the dedicated transporters in response to NA limitation is regulated by another histone modifier, the histone deacetylase Hst1. Interestingly, a lack of transporters again results in enhanced *EPA6* expression, implying a function in growth and adhesion during the infection process [39].

The *C. glabrata YPS* family comprising 11 cell wall genes is involved in interaction with host cells. The corresponding proteins share significant similarities with the *S. cerevisiae* yapsins (*YPS*). These GPI-anchored aspartyl proteases comprise five distinct proteins implicated in cell wall remodeling [41]. In *C. albicans*, secreted aspartyl proteases have been intimately associated with virulence [42]. The *C. glabrata* genes *YPS3* - *YPS11* are located in a specific gene cluster; expression of six cluster genes is induced after internalization by macrophages. Furthermore, *YPS1* and *YPS7* are implicated in cell wall integrity and cellular survival in stationary phase. Strains lacking the yapsins *YPS1* and *YPS7* or those lacking all eleven *YPS* genes, show attenuated virulence, implicating the *YPS* gene cluster in infectious processes [16]. Interestingly, the major adhesin Epa1 is stabilized in *Cgyps*Δ mutants, implying a direct or indirect role of the Yps proteases in Epa1 processing and/or proteolytic turnover. Consequently, *yps* gene deletion strains display increased adherence to epithelial cells [16].

## 3. Hypervirulence Factor *ACE2*

Exploiting a library of insertional signature-tagged mutants, the *C. glabrata* Ace2 transcription factor of the RAM (Regulation of Ace2 transcription factor and polarized Morphogenesis) network [43], has been identified as a hypervirulence factor [17]. The orthologous baker's yeast transcription factor Ace2 localizes only to the daughter cell nucleus, activates expression of early G1-phase genes, and mediates the separation of mother and daughter cells. Ace2 controls expression of a set of distinct cell wall target genes, including the chitinase *CTS1*, the putative glucanase *SCW11* and *DSE* genes implicated in the actual cell separation process [44,45]. Deletion of *ACE2* causes cell separation defects, resulting in pseudohyphal growth, clumping cells and detectable agar invasion [46].

Interestingly, the lack of the *C. glabrata* transcription factor Ace2 also causes cell separation defects, leading to the formation of large cell aggregates [17]. Strikingly, *ace2*Δ cells are hypervirulent in a neutropenic mouse infection model, causing 100% lethality after four days. The hypervirulent phenotype may, at least in part, be caused by drastically elevated proinflammatory cytokines due to abnormal exposure of fungal surface components in *ace2*Δ cells [17]. A proteomic analysis hints some 123 protein changes in the *ace2*Δ mutant. Consistent with expression data, morphogenesis and cell wall remodeling genes are down-regulated [18]. Notably, abundant cytoplasmic proteins are also detectable in the *ace2*Δ secretome, which are otherwise not found in the wild type supernatants [19]. These cytoplasmic proteins may increase immunogenicity, triggering an exacerbated immune response [18]. Noteworthy, the lack of *ACE2* changes the murine immune response only to *C. glabrata* but not to *C. albicans* mutants, which are rather attenuated in virulence [20]. In addition, the hypervirulent effect of *ace2*Δ cells is only observed in immunosuppressed mice [20]. Strikingly, while being highly pathogenic to humans, *C. glabrata* is very efficiently cleared when injected into immunocompetent mice. However, a number of inconsistencies exist in the literature concerning the use of mouse models for studying *C. glabrata* virulence. Since killing of wild type mice by systemic *C. glabrata* infections is at least a controversial issue, mice may better serve as *in vivo* systems to monitor growth, dissemination and colonization of organs and tissues [47].

## 4. Model Systems to Study Virulence of *C. glabrata*

Infection of mice with *C. glabrata* does not lead to the development of systemic candidiasis and subsequent death. Therefore, immunosuppressed mice are obtained by using 200 mg/kg cyclophosphamide administered three days before infecting with *C. glabrata*. This model system yields mortality rates of up to 100% after five days of infection without any evidence for necrosis or inflammation. However, high fungal burdens such as 2 × 10^8^ *C. glabrata* cells injected into the lateral tail vein are required, while lower burdens increase mouse survival [48]. This mouse model suggests the hypervirulence of *C. glabrata ace2*Δ cells [17,20], as well as the increased virulence of Pdr1 gain-of-function mutants [24]. As indicated above, survival of infected mice is not the best readout for *C. glabrata* virulence, since severe immunosuppression is required to obtain fungal killing. Nevertheless, immunocompetent mice have been successfully used to study the virulence or fitness phenotypes *in vivo* [16]. For example, for *YPS* deletion strains, the level of tissue colonization was used as a measure of fungal virulence [16]. Hence, quantifying organ colonization of *C. glabrata* strains in normal mice is a useful assay for fitness *in vivo* and thus directly relates to virulence [47].

In addition, *C. glabrata* virulence has been also investigated in a *Drosophila melanogaster* infection model. Whereas wild type flies survive the injection of 7,500 *C. glabrata* cells, *MyD88* mutant flies show strongly increased mortality [49]. Hence, this fly model could be used in the future to screen a larger number of *C. glabrata* strains for virulence phenotypes.

Another invertebrate model system used to study virulence of *C. albicans* and *Cryptococcus neoformans* is the Greater Wax Moth *Galleria mellonella* [50,51]. For survival assays, *C. albicans* cells are injected into the haemocoel of *G. mellonella* larvae. Notably, good correlations between virulence phenotypes in the invertebrate model and in murine models of systemic candidiasis exist [50,52]. Therefore, this convenient and inexpensive model system may be suitable to study virulence of *C. glabrata* on a large scale.

A *Caenorhabditis elegans* infection model has also been used to screen a library of 83 *C. albicans* transcription factor mutants for alterations in virulence. Five mutants were identified, two of which were previously shown to have defects in virulence in a murine model of candidiasis [53]. This model system may be suitable for high-throughput screening of putative antifungal compounds [54]. Whether this model system is also suitable to study *C. glabrata* virulence remains unclear.

## 5. Pigmentation as Virulence Factor

Many pathogenic fungi can produce pigments some of which are implicated in virulence [55,56,57]. Such pigments have diverse biological functions, including antioxidative effects [58,59], which counteract reactive oxygen species (ROS) produced by the host immune system to kill and eliminate invading microbial pathogens [60].

While *C. glabrata* was hitherto believed to be an unpigmented yeast species, recent work demonstrates the production of indole-derived pigments [61]. Pigment production requires the presence of tryptophan as the sole nitrogen source in the medium. Furthermore, the chemical composition was similar to the pigments produced by the lipophilic yeast *Malassezia furfur* [61], which are distinct from pigments produced by other pathogenic fungi via the melanin synthesis pathway [57]. Interestingly, pigment production by *C. glabrata* proceeds via the Ehrlich pathway [21], which mediates degradation of aromatic amino acids in *S. cerevisiae* [62]. Deletion of *ARO8* encoding an aromatic aminotransferase catalyzing a transamination reaction in the Ehrlich pathway, reduces pigmentation. In addition, *aro8*Δ mutants show increased susceptibility to H_2_O_2_ treatment. A similar phenotype was observed in wild type cells growing in non-pigment-inducing media. Furthermore, pigments may protect fungal cells against neutrophil attack, since a lack of pigments leads to killing hypersensitivity [21], suggesting a possible role for pigments in the survival of *C. glabrata* within the host. Similarly, pigmentation may also protect filamentous fungi like *Aspergillus fumigatus* from host killing [56].

## 6. Genome Plasticity and Tandem Repeats

Like the random chromosome alterations frequently observed in *C. albicans,* the *C. glabrata* genome of clinical isolates, although as yet not much appreciated, also appears to undergo chromosomal alterations, including chromosome loss, translocations and aneuploidy. The analysis of 40 clinical isolates shows drastic differences in their genome organization, suggesting a highly dynamic genome [63]. Chromosomal rearrangements, translocations, chromosome fusions and inter-chromosomal duplications lead to distinct karyotypes. Strikingly, *C. glabrata* is even able to perform *de novo* chromosome generation [63]. The authors speculate that the lack of a sexual cycle may cause tolerance to frequent chromosomal rearrangements, providing an explanation for the remarkable clonal population diversity. Interestingly, chromosomal rearrangements often occur at the same loci, as several isolates showed duplicated segments carrying genes associated with drug resistance (*CDR1, CDR2*) or survival in macrophages (*YPS* gene cluster [16]) [63]. This genomic plasticity of *C. glabrata* may therefore serve as a compensatory mechanism to allow for rapid adaptation to changing host conditions / environments and maybe compensate the absence of a functional sexual cycle.

These genome dynamics is also reflected in the high number of minisatellite sequences found in *C. glabrata* genomes [35]. Tandem repeats and selective domain amplifications are commonly found in both pathogenic and non-pathogenic yeast species, often occurring in adhesin or flocculation genes [35,64,65]. Notably, the majority of minisatellites are not conserved between baker's yeast and *C. glabrata*, although genes carrying minisatellites appear conserved. Remarkably, *C. glabrata* also harbors unusual types of minisatellites, so-called compound minisatellites with intermingled repeats and megasatellites containing long repeated motifs [35]. The evolutionary mechanism and origin of these minisatellites remains unclear, but a large number of *EPA* adhesion genes carry such repeats. A plausible hypothesis is that unusual minisatellites relate to high-frequency chromosomal rearrangements. This would further diversify expression as well as function of adhesion genes, which are considered important pathogenicity genes.

## 7. Phenotypic Switching of *C. glabrata*

Two distinct morphologies, core and irregular wrinkled, which result from high-frequency phenotypic switching mechanisms, were recently discovered in *C. glabrata*. The core system is composed of four phenotypes identified on the basis of their colony color on plates containing CuSO_4_. They are called white (Wh), light brown (LB), dark brown (DB) and very dark brown (vDB) [66,67]. In addition, cells of each of the core phenotypes can switch to the irregular wrinkled (IWr) phenotype and reverse back to core phenotypes. Most clinical isolates may undergo phenotypic switching, with DB being the most frequently observed species [67]. In addition, there are differences in the frequency of switching phenotypes depending on the sites of host colonization. For instance, vaginal isolates prefer DB, whereas genetically identical cells from the oral cavity were predominantly displaying the Wh phenotype [68]. These results strongly suggest roles for phenotypic switching in the adaptation to different host niches. Indeed, after injecting a mixture of DB and Wh or DB and IWr in a 50:50 ratio into a mouse model of systemic infection, mainly DB species appear in the spleen, liver and kidney after plating organ homogenates [69]. The outcome is similar for all organs, suggesting an advantage of DB cells over other switching phenotypes within the host. Importantly, the observed advantage of DB cells over Wh cells is not caused by increases in switching towards the DB phenotype, but rather arises from preferred organ colonization, as demonstrated by GFP-tagging of either the DB or the Wh cells in the injection mixture [69]. Different host niches may favor other switching phenotypes than DB. Although the molecular mechanisms underlying phenotypic switching in *C. glabrata* are enigmatic, switching might be important in *C. glabrata* infections and host colonization.

## 8. Resistance to Oxidative Stress and Survival Inside the Phagolysosome

The first encounters of *C. glabrata* with the host innate immune cells include phagocytic cells [70]. Remarkably, many pathogens have developed different strategies to escape from the phagosome following internalization. For example, *C. albicans* destroys macrophages by switching to the hyphal growth while *C. neoformans* can lyse macrophages or escape via phagosomal extrusion [71,72]. To date, little is known how *C. glabrata* responds to host cell phagocytosis and how it can survive and persist inside the phagolysosome. The lack of morphogenesis does not allow physical killing of host cells by *C. glabrata*. Moreover, phagolysosome maturation brings a hostile environment for pathogens, including hydrolytic enzymes as well as a lower pH due to acidification [73,74]. In addition, pathogen adhesion triggers extracellular host-derived ROS to kill pathogens [60]. Therefore, antioxidant activities seem plausible virulence factors in different pathogenic fungi [75-77]. For example, *A. fumigatus* lacking catalases normally degrading H_2_O_2_, shows attenuated virulence in a rat model of invasive aspergillosis [78]. For *C. glabrata*, a lack of *CTA1*, the gene encoding the only catalase, results in hypersensitivity to H_2_O_2_. Interestingly, *C. glabrata* strains show higher peroxide resistance than *S. cerevisiae* or *C. albicans,* suggesting a high intrinsic resistance to oxidative stress [23]. Indeed, Cta1 expression is induced after phagocytosis and both the number of peroxisomes and Cta1 localization to peroxisomes is enhanced [22]. Notably, peroxisome numbers decrease after prolonged residence of *C. glabrata* in the phagolysosome, perhaps via autophagy, to help *C. glabrata* surviving in the nutrient-limited environment. Deletion of *ATG11* or *ATG17* results in defects in the reduction of peroxisomes and reduced survival upon phagocytosis. Thus, in addition to surviving ROS attacks, recycling of potential internal nutrients or the use of host-derived nutrients may be beneficial for the persistence and survival of *C. glabrata* in the host after phagocytosis.

## 9. Mechanisms of Antifungal Resistance in *C. glabrata &* Modulation of Drug Susceptibility

For space constraints, we shall limit the discussion on antifungal drug resistance, but would like to refer to numerous excellent recent reviews on the use of antifungal drugs and the mechanisms of antifungal resistance in fungal pathogens [7,79-81]. It has been widely recognized that *C. glabrata* displays inherently high resistance to several antifungal drugs, limiting the efficacy of some antifungal drugs used in clinical therapy [7,79-81].

**Figure 1:**
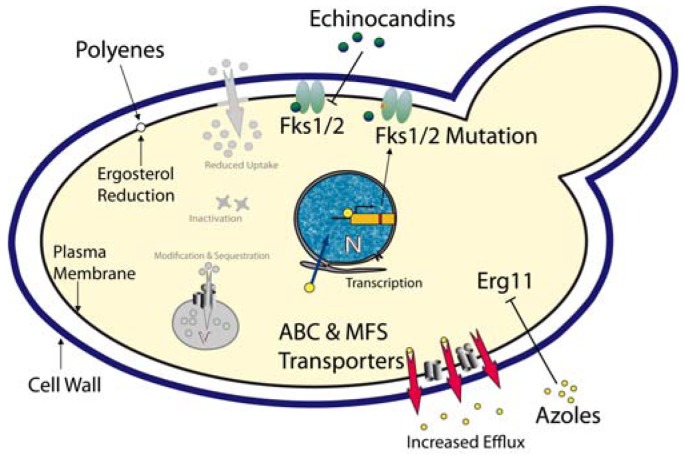
Prevalent antifungal drug resistance mechanisms in *Candida glabrata*. The cartoon depicts the major principal mechanisms causing antifungal drug resistance in *C. glabrata*, and indicates the actual drug targets.

***Azoles*** – These compounds represent most widely used class of antifungal drugs and they have been used to treat fungal infections for several decades. The cellular target of the azoles is the lanosterol 14-α-demethylase, encoded by the *ERG11* gene [82]. Inhibition of this enzyme efficiently blocks ergosterol biosynthesis, an essential fungal membrane component (Figure 1). When compared with other *Candida* spp, *C. glabrata* shows an inherently reduced azole susceptibility. In addition, prolonged and prophylactic treatment with azoles often results in the emergence of clinically resistant *C. glabrata* strains. For *C. albicans,* one azole resistance mechanism is the overexpression or mutation of the azole target Erg11 [83,84]. However, in *C. glabrata* azole-resistant clinical isolates, neither overexpression nor *ERG11* mutations seem to mediate resistance [85-87]. However, the transcriptional induction and massive up-regulation of drug efflux pumps, especially members of the ABC (ATP-binding cassette) transporter family and the major facilitator family efficiently prevent intracellular azole accumulation [88-91]. Three ABC transporters are involved in *C. glabrata* azole resistance: Cdr1, Cdr2 (Pdh1) and Snq2 [25,92,93].

Aus1, another fungal ABC transporter implicated in sterol uptake in yeast [94], may be somehow involved in a low intrinsic susceptibility of *C. glabrata* to azoles but the mechanisms behind remain unclear. The growth-inhibition by fluconazole is suppressed by the addition of serum to the medium, perhaps because *C. glabrata* can take up cholesterol from the serum under these conditions [95]. In addition, this effect was dependent on the presence of Aus1, implying that Aus1 might also be involved in the uptake of sterols in *C. glabrata* [96]. The authors propose that Aus1-mediated uptake of cholesterol from the medium can rescue the lack of membrane ergosterol as caused by Erg11 inhibition. This might contribute to the low susceptibility of *C. glabrata* to azoles.

***Polyenes*** - Polyenes are fungicidal antifungals and have been used for more than 50 years. These substances intercalate into ergosterol-containing membranes (*i.e.* mostly plasma membrane), thereby forming pores which result in leakage of cellular components, collapse of ion and electrical gradients and ultimately lead to cell death (Figure 1). Unfortunately, adverse side effects such as severe nephrotoxicity to the host rather than resistance make a long-term use of this class of antifungals difficult [97]. Resistance or decreased susceptibility to amphotericin B, the most prominent polyene, was reported in clinical isolates of different *Candida* spp including *C. glabrata* [98]. A reduction of the ergosterol content in the plasma membrane appears to correlate with reduced susceptibilities to amphotericin B [99].

***Echinocandins*** - The echinocandin antifungals are inhibitors of the Fks1/Fks2 1,3-β-D-glucan synthases, the enzymes responsible for synthesis of 1,3-β-d-glucan, a major and essential cell wall component of all fungi [100]. The approved candin drugs (caspofungin, anidulafungin and micafungin) are non-competitive Fks1/Fks2 inhibitors, disrupting the integrity and structural organization of the cell wall, thereby exerting fungicidal action [101]. As expected, mutations in *FKS1* and *FKS2* encoding the catalytical subunits of the 1,3-β-d-glucan synthases mediate echinocandin resistance in *C. glabrata* [27-29] (Figure 1). Some 11 new mutations detected in *FKS1* or *FKS2* of *C. glabrata* clinical isolates cause reduced susceptibility to echinocandins. However, reduced enzymatic activities of Fks1/2 mutant variants might affect fitness in the host and therefore promote low frequency of echinocandin resistance [30]. Notably, ectopic overexpression of the Cdr2 ABC transporter causes efflux-mediated tolerance to caspofungin in *C. albicans* laboratory strains, as well as in clinical isolates [102].

Taken together, a composite multidrug resistance phenotype is often caused by the parallel or consecutive activation of a number of distinct mechanisms operating in all living cells from bacteria to cancer cells [103-106]. Thus, while mechanisms such as reduced drug uptake, intracellular catabolism, target gene mutations, overexpression and gene amplification, signaling and stress response pathways, membrane lipid changes, vacuolar sequestration operate in most infectious microbes, clinical resistance in *C. glabrata* patient isolates may result mainly from transporter-mediated drug efflux.

Finally, several mechanisms are operating in *C. glabrata* to modulate antifungal resistance phenotypes. The Zn(2)-Cys(6) transcription factor Pdr1 controls the expression of at least three ABC transporters involved in azole resistance in *C. glabrata* [25,86]. Pdr1 may directly bind antifungal drugs or other xenobiotics to become transcriptionally active and trigger transcription of drug efflux pumps leading to multidrug resistance [107]. As in the non-pathogenic baker's yeast, gain-of-function mutations in *PDR1* result in a constitutively active transcription factor, and these are prevalent in the majority of azole-resistant clinical isolates. In addition, strains harboring constitutively active *PDR1* alleles also show increased virulence in mice, even in the absence of azole treatment [24]. These results strongly suggest that Pdr1 may account for most cases of clinical azole resistance in *C. glabrata*. Notably, similar to yeast, Pdr1 may also mediate azole resistance in response to mitochondrial dysfunction in *C. glabrata* [108-110].

Interestingly, chromatin modification may account for novel mechanisms mediating drug resistance in certain fungi. For example, in *S. cerevisiae,* deletion of *RPD3* encoding a histone deacetylase also results in enhanced susceptibility to azoles. Furthermore, Rpd3 is required for up-regulation of an ABC transporter in response to cycloheximide [111]. In addition, treatment of *C. albicans* with histone deacetylase inhibitors also strongly increases the sensitivity of fungal pathogens to different classes of antifungal agents [112,113]. Notably, induction of the ABC transporters *CDR1* and *CDR2* upon fluconazole treatment is significantly reduced in the presence of the histone deacetylase inhibitor trichostatin A [112]. Hence, histone-modifying enzymes might also be involved in the regulation of drug resistance in *C. glabrata* and should be considered as potential future drug targets.

## 10. Conclusions and Perspectives

*C. glabrata* is a very successful human pathogen, accounting for up to 25% of all clinical *Candida* infections. Although new insights concerning the molecular mechanisms mediating virulence are surfacing, many open questions concerning host adaptability and pathogenicity remain. Hence, the field needs as much as possible genome-wide and global approaches, whereby researchers can make use of tools such as a deletion collection, an overexpression collection or an epitope-tagged ORFome just to name a few tools that have been revolutionizing research in the non-pathogenic baker's yeast [114-116]. Importantly, we need to have a catalogue of potential virulence genes, similar to attempts initiated for *C. albicans* or *Cryptococcus neoformans* [117,118]. However, to exploit such a tool, the community needs to develop appropriate mammalian models of virulence, enabling studies on commensalism, immune evasion, colonization, host dissemination and pathogenic conversion. Furthermore, there is a need for a quantitative understanding of the dynamic interplay of mammalian hosts with fungal pathogens. Deep-sequencing [119,120] as well as single cell imaging will be highly beneficial to understand invasion and dissemination in the host. Comparative functional genomics is taking advantage of the comparison with the non-pathogenic and well-studied relative *S. cerevisiae,* hoping for new insights. The genome organization show rather limited differences, some of which may suffice to explain the striking differences in virulence. However, the remarkable and nearly endless combinatorial complexity of genetic interactions [121] will make it difficult to come up with meaningful cause-consequence conclusions based on small genomic or genetic differences identified by comparing *S. cerevisiae* and *C. glabrata*.

Hence, the future calls for integration of different disciplines, including mathematics and molecular approaches delivering quantitative data. The use of systems biology approaches such as predictive mathematical modeling of quantitative biological data will facilitate a better understanding of the complexity underlying fungal pathogenicity. Furthermore, diagnostic tools exploiting molecular methods will have to improve concerning both speed and reliability, since this will facilitate clinical therapy. Of course, the clinical day-to-day reality is always in need for new and more efficacious antifungal drugs once the persistent difficulties concerning the rapid and accurate diagnosis of fungal species causing diseases have been overcome. In addition to classical small molecule drugs, novel antibody-based approaches may aid both diagnostic and therapeutic approaches, and even vaccines are now entering stage as feasible approaches to cure or combat systemic fungal disease.

## Figures and Tables

**Table 1:** *Candida glabrata* genes implicated in pathogenicity and virulence.

**Gene**	**Deletion phenotype**	**References**
*EPA* gene family	Reduced adherence, organ colonization and biofilm formation	[11,12,13,14,15]
*SIR3, RIF1*	Increased adherence and kidney colonization	[12]
*YPS* gene family	Reduced organ colonization, increased adherence	[16]
*ACE2*	Hypervirulence, cell separation defect	[17,18,19,20]
*ARO8*	Reduced pigmentation, increased susceptibility to oxidative stress	[21]
*CTA1*	Increased susceptibility to oxidative stress	[22,23]
*ATG11, ATG17*	Reduced survival upon phagocytosis	[22]
*PDR1*	Increased azole susceptibility; GOF mutations: increased virulence and organ colonization, azole resistance	[24]
*CDR1, CDR2, SNQ2*	Reduced azole resistance	[25,26]
*FKS1, FKS2*	Mutations lead to echinocandin resistance	[27,28,29,30]
